# Energy balance components in persons with paraplegia: daily variation and appropriate measurement duration

**DOI:** 10.1186/s12966-017-0590-z

**Published:** 2017-09-26

**Authors:** Tom E. Nightingale, Sean Williams, Dylan Thompson, James L. J. Bilzon

**Affiliations:** Department for Health, University of Bath, Bath, BA2 7AY UK

**Keywords:** Energy balance, Spinal cord injury, Energy intake, Energy expenditure, Physical activty, Diet, Measurement, Paraplegia, Intra-individual variance, Assessment

## Abstract

**Background:**

Despite obesity being highly prevalent in persons with spinal cord injury (SCI), our current understanding of the interactions between energy balance components, which may contribute to this, is limited. The primary aim of this study is to identify the intra-individual variability of physical activity dimensions across days and suggest an appropriate monitoring time frame for these constructs in adults with SCI. The secondary aim is to examine these parameters with regard to energy intake and dietary macronutrient composition.

**Methods:**

Participants [33 men and women with chronic (> 1 year post injury) paraplegia; age = 44 ± 9 years (mean ± S.D.] wore an Actiheart™ PA monitor and completed a weighed food diary for 7 consecutive days. Spearman-Brown Prophecy Formulae, based on Intraclass Correlations of .80 (acceptable reliability), were used to predict the number of days required to measure energy balance components. Linear mixed-effects analyses and magnitude-based inferences were performed for all energy intake, expenditure and physical activity dimensions. Adjustments were made for age, injury level, wear time, sex, day of the week and measurement order as fixed effects.

**Results:**

To reliably measure energy expenditure components; 1 day [total energy expenditure (TEE)], 2 days [physical activity energy expenditure (PAEE), light-intensity activity, moderate-to-vigorous PA (MVPA)], 3 days [physical activity level (PAL)] and 4 days (sedentary behaviour) are necessary. Device wear time (*P* < 0.02), injury level (*P* < 0.04) and sex (*P* < 0.001) were covariates for energy expenditure components. Four and ≤24 days are required to reliably measure total energy intake (kcal) and diet macronutrient composition (%), respectively. Measurement order (from day 1–7) was a covariate for total energy intake (*P* = 0.01).

**Conclusions:**

This is the first study to demonstrate the variability of energy intake and expenditure components in free-living persons with chronic (> 1 year) paraplegia and propose suitable measurement durations to achieve acceptable reliability in outcome measures. Device wear time and measurement order play a role in the quality of energy expenditure and intake data, respectively, and should be considered when designing and analysing studies of energy balance components in persons with SCI.

**Trial registration:**

N/A

## Background

Paraplegia is a condition characterised by impaired motor or sensory function of the lower extremities, with paralysis commonly caused by spinal cord injury (SCI). Persons with chronic (> 1 year) paraplegia perform low levels of physical activity [1] and have more adipose tissue for any given age in comparison to non-disabled controls [2]. As such, individuals with SCI are at increased risk of developing chronic diseases, such as type 2 diabetes and cardiovascular disease, compared to the general population [3–5]. As changes in body mass are linked to an imbalance between energy intake and energy expenditure [6], energy balance can be expressed as: *energy balance = energy intake – energy expenditure*.

Energy intake is composed of the three macronutrient food groups, carbohydrate, protein, and fat, plus alcohol. Total energy expenditure (TEE) can be partitioned into resting metabolic rate (RMR), diet-induced thermogenesis (DIT) and, physical activity energy expenditure (PAEE). In absolute terms, all three TEE components are lower in persons with SCI, contributing to lower TEE [7–9]. RMR is lower due to atrophy of lower extremity fat free mass, as a result of lower limb paralysis, and impaired innervation of the sympathetic nervous system [10]. PAEE is lower due to mobility impairments and the use of a smaller available muscle mass (upper-body), while hormonal and body composition changes post SCI likely impact upon DIT [11]. If energy intake increases slightly or remains constant for a prolonged period, concurrent reductions in TEE can lead to a sustained energy surplus, which drives the notable trajectory of weight gain following SCI [12]. PAEE is the most important component of TEE due to its high variability amongst free-living individuals and is therefore potentially more malleable [13]. PAEE can be further partitioned into energy expended via light-intensity or moderate-to-vigorous intensity physical activity (MVPA). In the general population, the dose and intensity of physical activity are important considerations for reducing the risk of all-cause mortality [14, 15]. Sedentary behaviour [any behaviour ≤1.5 metabolic equivalents (METS), i.e. sitting or lying down] is now also viewed as an important lifestyle behaviour, distinct from physical activity, due to its independent effects on cardiometabolic health [16].

The advancements in multi-sensor technologies now allow researchers to capture these physical activity dimensions with high resolution, specifically in persons who use wheelchairs [17]. However, measuring components of habitual free-living energy expenditure and physical activity dimensions is made difficult due to considerable intra-individual variability, some of which is related to natural variance in human behaviour and some related to unexplained random error, which is related to the validity and reliability of methods. Studies in non-disabled participants have suggested a range of measurement periods are necessary to accurately and reliably quantify MVPA (2–10 days) and sedentary behaviour (3–8 days) [18–21]. Error in any measurement gives rise to unexplained variance, either attenuating or enhancing ‘true’ relationships between these outcomes and population health. Therefore, in order to better understand the role of various physical activity dimensions on cardiometabolic disease in this at risk population, it is important to appreciate their day-to-day variation. To measure the impact of an exercise intervention in persons with SCI we recently encouraged researchers to monitor free-living energy balance [22]. This permits the assessment of behaviours that might erode the effectiveness of such interventions, i.e. compensatory increase in energy intake or substitution of existing physical activity [23]. Determining the amount of days necessary to reliably measure components of energy balance may inform future study designs and data reduction strategies in this population.

Consequently, the primary aim of this study is to identify the intra-individual variability across days, and suggest an appropriate monitoring time-frame for estimating habitual physical activity dimensions in adults with SCI using a multi-sensor device. The secondary aim is to examine these parameters with regard to energy intake and macronutrient composition, calculated using weighed diet recalls over a corresponding 7-day period.

## Methods

### Participants and eligibility criteria

Thirty-three participants with chronic paraplegia were enrolled as part of two independent research trials, whereby habitual lifestyle behaviours were measured in community dwelling individuals with SCI [24, 25]. Participants were included if they had a chronic (> 1 year) spinal cord lesion below the first thoracic vertebrae, were between 18 and 65 years of age and were considered weight stable (± 3 kg) for the preceding 3 months with no plans to change their diet or exercise behaviours. Volunteers with neurologically incomplete injuries were considered eligible if they were wheelchair users for more than 75% of their waking day. Participants with active medical issues (pressure sores, urinary tract infection or cardiac disorders) were excluded from the study. Ethical approval for these trials was granted by the University of Bath’s Research Ethics Approval Committee for Health (REACH) and the South West (Exeter) National Research Ethics Service Committee (REC reference number 14/SW/0106). Each participant provided signed and informed consent prior to taking part in the trials. Participants wore a multi-sensor activity monitor (Actiheart™, Cambridge Neurotechnology Ltd., Papworth, UK) and completed a weighed food diary for 7 consecutive days following an initial laboratory visit. During this period, participants were asked not to alter their normal activity patterns or dietary behaviours.

### Body composition and anthropometrics

Each participant was asked to void their bladder prior to body mass measurement, which was taken with participants wearing light clothing, using platform wheelchair scales (Detecto® BRW1000, Webb City, MO, USA). The wheelchair, along with participants’ shoes were weighed separately and subtracted to derive an accurate body mass for each participant. Supine height was measured in centimetres along the left hand side of the body using a non-elastic tape measure (Lufkin, Sparks, MD, US). American Spinal Injury Association Impairment Scale (AIS) was self-reported.

### Resting metabolic rate

Resting metabolic rate (RMR) was measured around 08.00 am after an overnight fast (> 10 h) via indirect calorimetry, in accordance with best practice guidelines [26]. Participants were asked to abstain from strenuous exercise, caffeine (tea/coffee) and alcohol for twenty-four hours prior to visiting the laboratory. Following a 20-min rest in a supine position, expired air was collected into four 200-L Douglas Bags (Hans Rudolph, Kansas City, MO, USA) over a 20-min period. Oxygen and carbon dioxide concentrations were measured from a known volume of each sample using paramagnetic and infrared analysers (MininMP 5200, Servomex Ltd., Sussex, UK), calibrated according to manufacturer’s instructions prior to use. Participants also wore a Polar T31 heart rate monitor (Polar Electro Inc., Lake Success, NY, USA) during RMR to determine resting heart rate.

### Energy expenditure assessment

Participants wore an Actiheart™, which was fitted using two adhesive ECG chest electrodes (Telectrode T815, Bio-Protech Inc., Exeter, UK) according to manufacturer’s instructions, for 7 consecutive days. The device integrates uniaxial accelerometry and heart rate signals using branched model equations to estimate energy expenditure. This has been described in more detail previously [27, 28]. Individual heart rate calibration of this device has been shown to improve energy expenditure prediction in non-disabled individuals [29–33] and wheelchair users [24]. Therefore, to individually calibrate this device, the relationship between energy expenditure and heart rate in each participant was determined. This was achieved through an incremental exercise test performed on an arm-crank ergometer (Lode Angio, Groningen, Netherlands) during the initial laboratory visit. Resistance was increased by ~14 W every 3-min until the point of volitional exhaustion. Continuous gas exchange measurements were collected using a TrueOne® 2400 computerised metabolic system (ParvoMedics, Salt Lake City, UT, USA). Energy expenditure and corresponding heart rate data were averaged over the final minute of each 3-min stage. Data collected at rest and during this exercise test (designed to cover a range of submaximal/maximal exercise intensities) can be entered into the Actiheart™ software to generate an individual heart rate calibration specific to each participant. As common equations to predict RMR in the general population over-predict RMR for persons with SCI [10], measured RMR (via indirect calorimetry) was also entered into the Actiheart™ software. In our study sample, the predicted RMR from the Actiheart™ software (derived from body mass using the Schofield eq. [34]) resulted in 14 ± 11% over-prediction compared to measured RMR. Energy expenditure is presented as total energy expenditure (TEE), and its constituent parts; RMR, diet-induced thermogenesis (DIT) and physical activity energy expenditure (PAEE). DIT was 10% of daily TEE derived from the Actiheart™. Data were also expressed as various dimensions of physical activity behaviours; physical activity level (PAL; TEE/RMR) and minutes per day spent in different intensities of activities on the basis of metabolic equivalents (METs); sedentary <1.5 METs, light- 1.5 – 2.9 METs and moderate-to-vigorous (MVPA)- >3.0 METs.

### Energy intake assessment

Participants were asked to keep a detailed record of their food and fluid intake for a ‘typical’ and continuous 7-day period. Each participant received a set of weighing scales (PL11B Digital Scale, Smart Weigh, Chestnut Ridge, NY, USA), and were trained by researcher how to accurately weigh and record foodstuffs. Weighed food records are a more valid measure of energy intake than other dietary recall methods [35]. Participants were instructed not to alter their dietary patterns during the monitoring period. If weighing of foodstuffs was not possible or overly burdensome when eating out for example, participants were asked to provide as much information about the meal as possible (i.e. name of restaurant, what they ordered and a rough estimate of size). Diet records were analysed using Nutritics Professional Nutrition Analysis Software (Nutritics Ltd., Dublin, Ireland), to calculate daily energy intake (kcal) and percentage of macronutrients (protein, fat, carbohydrate and alcohol).

## Statistical analyses

All estimations were made with R (Version 3.3.1, R Foundation for Statistical Computing, Vienna, Austria). To determine the reliability of energy intake and expenditure measures, Intraclass Correlation Coefficients (ICC) were obtained from linear mixed-effects models [36] via the *psychometric* package [37]. Dependent variables were log transformed before modelling [38], and then effects and standard deviations were back-transformed to percentages. Separate analyses were performed for each energy intake and expenditure measure. The random effect was subject identity, whilst fixed effects were used to adjust for age, injury level, wear time, sex, and day of week. Additionally, the linear effect of ‘day of entry’ was used to evaluate any change in energy intake reporting practices, estimated energy expenditure and physical activity dimensions across the seven days of monitoring. To constitute a ‘valid’ day, a wear-time cut-off of 80% (> 1152 min per day) was used as this has been adopted previously when using multi-sensor devices [25, 39]. The ICC values describe the ratio of the between subject variance to the total variance [between subject variance/(between subject variance + residual variance)] [40]. The number of days needed to obtain a reliability of 0.80 was estimated using the Spearman Brown prophecy formula [41, 42]: N = ICC_t_/(1-ICC_t_)*[(1-ICC_s_)/ICC_s_], where N = number of days needed, ICC_t_ = desired level of reliability, and ICCs = reliability for single days. As recommended by Baranowski and de Moor [43], an ICC of 0.80 was chosen as the cut-off for acceptable reliability. Coefficient of variation (CV) was calculated to also explain intra-individual variability ((SD/mean)*100) between days of the week.

Magnitude-based inferences were used to provide an interpretation of the real-world relevance of the outcomes [44]. The modifying effects of covariates were calculated, either as differences between levels of a categorical covariate (e.g. male vs female) or as the change associated with a two standard deviation (SD) increase in a numeric covariate [45]. That is, numeric covariates were evaluated as the difference between a ‘typically low’ value (i.e., 1 S.D. below the mean) verses a ‘typically high’ (i.e., 1 S.D. above the mean) value for the covariate, which enables the magnitude of the effect to be compared directly across different numeric covariates. A value equivalent to a standardised difference in means of 0.20 was set as the smallest worthwhile effect threshold. Effects were classified as unclear if the percentage likelihood that the true effect crossed both positive and negative smallest worthwhile effect thresholds were both greater than 5%. Otherwise, the effect was deemed clear, and was qualified with a probabilistic term using the following scale: <0.5%, most unlikely; 0.5–5%, very unlikely; 5–25%, unlikely; 25–75%, possible; 75–95%, likely; 95–99.5%, very likely; >99.5%, most likely [46].

## Results

Participant characteristics are described in Table 1. The Actiheart™ was worn for 7 days, with valid days (> 80% wear time over 24 h) ranging from between 4 and 7 days. Across valid days, the Actiheart™ was well tolerated; 96 ± 3% wear time each day. Actiheart™ technical issues (*n* = 2) and a misplaced food diary (*n* = 1) resulted in exclusion of participant data for the energy expenditure (*n* = 31) and intake analyses (*n* = 32), respectively.

**Table 1:** Participant characteristics

Characteristic	Mean ± SD *(range)*	
Age (years)	44 ± 9 *(22–61)*	
Level of injury	T7 *(T1 – L4)*	
AIS classification A and B	A = 28 (85%)	B = 5 (15%)
Time since injury (years)	15 ± 10 *(1–39)*	
Body mass (kg)	76.1 ± 12.5 *(54.2–99.6)*	
Sex	Female = 6 (18%)	Male = 27 (82%)
PAL	1.40 ± 0.15 *(1.20–1.85)*	

*AIS* American Spinal Injury Association Impairment Scale, *PAL* physical activity level

## Covariates

### Wear time

When all data were analysed without excluding invalid days (wear time < 80%), there was a significant main effect of wear time for TEE (*P* = 0.02), PAEE, (*P* < 0.001), PAL (*P* = 0.003), sedentary time (*P* = 0.017) and light-intensity activity (*P* = 0.003) with inferences of ‘likely trivial’, ‘very likely’, ‘likely’, ‘possibly’ and ‘likely’ substantial differences, respectively. When the Actiheart™ was worn for a greater percentage of the day, the estimated energy expenditure values (except for sedentary time which was the opposite) were higher. When this analysis was performed only including valid days (wear time > 80%), this covariate only had a ‘trivial’ impact on energy expenditure variables (*P* > 0.47).

### Level of spinal cord injury

Injury level was a significant covariate for energy expenditure variables; TEE (*P* = 0.03), PAL (*P* = 0.04), sedentary time and MVPA (both, *P* = 0.03), all with inferences of ‘likely’ substantial differences. The difference/effect (lower and upper 95% CI) of a 2 SD change in injury level (lower lesion) was associated with increased TEE; 12% (1, 25%), PAL; 6% (0, 12%), MVPA; 95% (6, 261%) and, reduced sedentary time; −8% (−14, −1%).

### Sex

Sex was also a significant covariate for energy expenditure variables; TEE, PAEE, PAL, sedentary time, light and MVPA (all, *P* < 0.001), with an inference of ‘most likely’ substantial differences between males and females. Females were less active and more sedentary than males. With regards to energy intake variables, females consumed less energy (total kcal) than males (*P* = 0.03), inference; ‘likely’ substantial difference.

### Order (1st to 7th day of measurement)

There was a main effect of order (from day 1–7) for total energy intake (*P* = 0.01), with the change across the course of one week (1st to 7th day of measurement) associated with decreased estimated total energy intake, −15% (−24, −4%) with an inference of ‘possibly’ substantial difference. There was no effect of order on energy expenditure variables (*P* > 0.23) or diet macronutrient composition (*P* > 0.70) with possibly ‘trivial’ or ‘unclear’ inferences.

## Day-to-day variation

The mean (95% CI) intra-individual variability (CVs) for each energy expenditure and intake outcome are shown in Table 2. Energy expenditure (sedentary, light-intensity, MVPA) and intake variables (macronutrient composition) that were ‘possibly’ or ‘likely’ influenced day of the week are shown in Table 3. Alcohol intake was significantly higher for Friday and Saturday compared to Monday, Wednesday and Thursday. The effect of day of the week was ‘unclear’ for TEE, PAEE, PAL and total energy intake.

**Table 2:** Intra-individual variability in energy intake and expenditure outcomes

Outcome	Mean intra-individual CV (95% CIs)
*Energy expenditure*	
TEE (kcal·d^−1^)	7% (6–8%)
PAEE (kcal·d^−1^)	34% (29–40%)
PAL	7% (5–8%)
*Physical activity dimensions*	
Sedentary time (min∙d^−1^)	9% (7–11%)
Light-intensity (min∙d^−1^)	46% (33–60%)
MVPA (min∙d^−1^)	97% (76–117%)
*Energy intake*	
Total (kcal·d^−1^)	26% (22–30%)
Protein (%)	26% (22–31%)
Fat (%)	26% (19–32%)
Carbohydrate (%)	20% (17–23%)
Alcohol (%)	160% (131–188%)

*CIs* confidence intervals, *CV* coefficient of variation, *MVPA* moderate-to-vigorous physical activity, *PAEE* physical activity energy expenditure, *PAL* physical activity level, *TEE* total energy expenditure

**Table 3:** Mean daily energy expenditure and intake variables with outputs from linear mixed-effects models and magnitude based inferences

	Energy expenditure variables			Energy intake variables			
	Sedentary; <1.5 METs (min·d^−1^)	Light-intensity; 1.5–2.9 METs (min·d^−1^)	MVPA; ≥ 3 METs (min·d^−1^)	Protein (%)	Fat (%)	Carbohydrate (%)	Alcohol (%)
Monday	1282 (1220, 1345)	154 (96, 211)	4 (0, 19)	20.0 (17.1, 22.8)	32.7 (29.3, 36.1)	45.6 (41.2, 50.0)	1.8 (0.0, 4.8)
Tuesday	1237 (1175, 1299)	188 (131, 245)	16 (1, 30)	19.0 (16.2, 21.9)	34.4 (30.1, 37.8)	44.3 (39.9, 48.7)	2.5 (0.0, 5.4)
Wednesday	1274 (1210, 1338)	159 (100, 218)	7 (0, 22)	18.3 (15.4, 21.1)	35.7 (32.3, 39.1)	45.0 (40.6, 49.4)	1.1 (0.0, 4.1)
Thursday	1281 (1219, 1343)	147 (90, 204)	12 (0, 26)	21.0^a,b,c^ (18.2, 23.9)	34.7 (31.3, 38.1)	42.7 (38.3, 47.2)	1.7 (0.0, 4.7)
Friday	1235 (1174, 1296)	196 (140, 252)	10 (0, 24)	17.1 (14.2, 19.9)	32.1 (28.7, 35.6)	45.9 (41.5, 50.3)	4.9 ^d,e,f^ (1.9, 7.9)
Saturday	1242 (1180, 1303)	176 (119, 232)	23 (8, 37)	18.4 (15.5, 21.3)	35.1 (31.7, 38.6)	41.5 (37.1, 45.9)	4.9 ^g,h,i^ (1.9, 7.9)
Sunday	1241 (1179, 1304)	185 (127, 242)	14 (0, 29)	19.2 (16.3, 22.0)	35.9 (32.5, 39.4)	42.8 (38.3, 47.2)	2.3 (0.0, 5.2)
Mean	1255 (1207,1304)	172 (127, 216)	12 (1, 24)	19.0 (16.7, 21.3)	34.4 (32.1, 36.7)	44.0 (40.6, 47.3)	2.7 (0.4, 5.0)
*P*-value	0.26	0.33	0.22	0.11	0.23	0.20	0.05
Inference	‘Possibly’ substantial difference	‘Possibly’ substantial difference	‘Possibly’ substantial difference	‘Likely’ substantial difference	‘Likely’ substantial difference	‘Possibly’ substantial difference	‘Likely’ substantial difference
Effect of highest-lowest day (%)	4.6 (−3.6, 12.7)	21.4 (−22.5, 65.3)	43.4 (−27.2, 114.1)	18.9 (−0.8, 38.6)	13.2 (−3.4, 29.8)	9.9 (−5.4, 25.2)	60.1 (−0.5, 120.7)

Values are means (95% CIs). ^a^different to Wednesday (*P* = 0.027), ^b^different to Friday (*P* = 0.008), ^c^different to Saturday (*P* = 0.033), ^d^different to Monday (*P* = 0.046), ^e^different to Wednesday (*P* = 0.016), ^f^different to Thursday (*P* = 0.039), ^g^different to Monday (*P* = 0.045), ^h^different to Wednesday (*P* = 0.015), ^i^different to Thursday (*P* = 0.039)

*METs* metabolic equivalents, *MVPA* moderate-to-vigorous physical activity

## Appropriate measurement duration

**Fig. 1:**
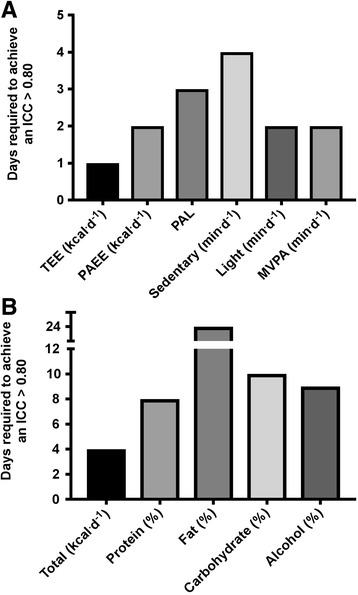
Number of monitoring days necessary to achieve acceptable reliability (ICC > 0.80) in various energy expenditure (**a**) and intake (**b**) variables. ICC, intra-class correlation; MVPA, moderate-to-vigorous physical activity; PAEE, activity energy expenditure; PAL, physical activity level; TEE, total energy expenditure

The number of days required to reliably (ICC > 0.80) estimate energy expenditure variables ranged from 1 (TEE) to 4 (sedentary behaviour) days (Fig. 1a). Whereas, between 4 (total energy intake) to 24 (fat) days were predicted to reliably estimate energy intake variables (Fig. 1b).

## Energy balance

**Fig. 2:**
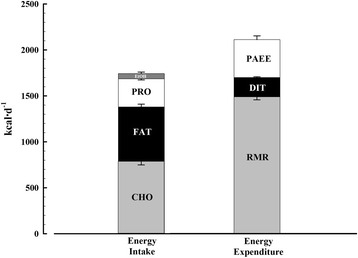
Components of energy balance under free-living conditions in individuals with chronic paraplegia. Values are means ± SEs (percentage that each component contributes to total daily energy intake and expenditure). CHO: carbohydrate, DIT: Diet-induced thermogenesis, EtOH: alcohol, PAEE: physical activity energy expenditure, PRO: protein, RMR: resting metabolic rate

Figure 2 presents the components of energy balance measured over 7 days under free-living conditions in persons with chronic paraplegia. For descriptive purposes, Energy intake (*n* = 32) was 1742 ± 72 kcal·d^−1^ [carbohydrate: 787 ± 38 kcal·d^−1^ (44%), fat: 592 ± 30 kcal·d^−1^ (34%), protein: 306 ± 13 kcal·d^−1^ (19%) and, alcohol: 57 ± 16 kcal·d^−1^ (3%). Energy expenditure (*n* = 31) was 2103 ± 74 kcal·d^−1^ [resting metabolic rate: 1481 ± 32 kcal·d^−1^ (70%), diet-induced thermogenesis: 211 ± 7 kcal·d^−1^ (10%) and, physical activity energy expenditure: 411 ± 42 kcal·d^−1^ (20%, range 6–36%).

## Discussion

These findings provide key insights into the daily variability of free-living physical activity energy expenditure and energy intake patterns in persons with SCI. The results indicate physical activity dimensions (sedentary, light and MVPA) and diet macro-nutrient composition vary habitually from day-to-day in this population, with ‘possible’ and ‘likely’ substantial differences between days of the week. Intra-individual daily variability (CVs) was large; ranging from 7 to 97% for energy expenditure variables and 20–160% for energy intake variables. Based on our results, anywhere between 1 (TEE) to 4 (sedentary) and 4 (total kcal) to 24 (Fat %) days of monitoring are necessary to achieve acceptable reliability when measuring energy expenditure and intake variables, respectively. These findings are likely dependent on the instruments and methodology used to capture energy balance components in this unique population (persons with chronic paraplegia). Furthermore, in this cohort, males and persons with lower spinal cord injury lesions were more active and less sedentary than females and persons with higher spinal cord injury lesions.

This is the first study to examine the daily variance and appropriate number of days necessary to reliably estimate physical activity behaviours in wheelchair users with SCI, with 2 days necessary for MVPA. Due to the potential logistical issues of administering and collecting devices; these days should likely be sequential. Physical activity constructs have been examined previously in non-disabled populations, with findings suggesting >3 weekdays are required to reliable measure MVPA using a multi-sensor device (Sensewear) in middle-aged non-disabled adults [19]. It has previously been suggested that at least 7 days were required to reliably estimate MVPA in younger (age; 31 ± 12 years) and very active (MVPA; 66 ± 28 min∙d^−1^) participants using a tri-axial accelerometer (GT3X+) [20]. In older adults, MVPA is generally planned, predictable and less variable [47]. Numerous psychosocial and environment barriers, for example fatigue, lack of accessible facilities and unaffordable equipment [48, 49] likely make it difficult for wheelchair users to spontaneously perform MVPA. This, coupled with lower levels of physical activity [mean MVPA (95% CIs): 12 (1, 24) min·d^−1^], could explain why fewer days are necessary to reliably measure MVPA in individuals with SCI.

Generally, within studies, more days are required to reliably measure sedentary behaviours, ranging from 3 to 8 days [18, 20, 21]. This is in keeping with the current study’s results. Sedentary behaviours are less predictable from day-to-day, as such more monitoring days are required to reliably predict this behaviour. These inconsistent recommendations are influenced by the type of activity dimension (i.e. MVPA or sedentary time) being measured, population characteristics (i.e. age, sex, physical activity level) and sensitivity of the measurement tool (i.e. multi-sensor, tri or uni-axial accelerometer or pedometer). This emphasises the need for researchers to establish the most appropriate monitoring time frame to reliably capture the specific physical activity dimension of interest, using particular measurement tools in their population of interest.

Only one day is required to reliably predict TEE, which is less variable from day-to-day as its largest component (~ 70%), RMR, is a constant. PAL (TEE/RMR), a normalised measure of AEE, also factors in the constant of RMR which explains why it is more ‘stable’ and requires fewer days to reliably quantify it than a measure of AEE (kcal∙d^−1^). Therefore, the approach to measurement should be different depending on the energy expenditure outcome of interest. Magnitude-based inferences suggest there are ‘possibly substantial differences’ between days for sedentary behaviour, light-intensity and MVPA. Consequently, day of the week should not be overlooked when designing and analysing data from physical activity monitoring studies that involve <7 days measurement in persons with SCI. This is consistent with findings in non-disabled populations, whereby differences in physical activity behaviours between days of the week and weekdays vs. weekend days have been observed [18, 19, 21]. Hence, the inclusion of a weekend day to reliably measure habitual sedentary behaviour, light-intensity activity and MVPA is justifiable based on the currently available evidence.

Another important consideration is selecting an appropriate wear time cut–off to represent a valid day. When all days were included in the analysis, wear time was a significant (*P* ≤ 0.02) covariate in the linear mixed effect models for TEE, PAEE, PAL, sedentary and light-intensity activity. Yet, when only valid days (>80% wear time, used previously for multi-sensor devices in two recent randomised controlled trials [25, 39]) were included in the analysis this covariate had a ‘trivial’ effect (*P* > 0.47) on energy expenditure variables. Aadland & Ylvisaker [20] demonstrated that reliability increased with stricter wear time criteria. Yet the strictest wear time criteria they applied was ≥12 h/day to represent a valid day, compared to ~19 h/day (80%) used in this current study. Furthermore, the actual Actiheart™ wear time was 23 h/day (range 83–99%) for valid days included in the analysis, which could explain the fewer measurement days required to reliably measure physical activity variables compared to studies with more lenient wear time criteria.

The energy intake and macronutrient composition reported herein is consistent with previous findings in persons with SCI [50–52]. The results of this current study demonstrate that 4 days is required to reliably measure total energy intake. Although weekend data were not included or alcohol intake reported, Gorgey et al.*,* [53] similarly found that 3 days were appropriate to measure energy intake with weighed food diaries in this population. While reliable, questions have been raised about the validity of self-reported energy intake [54], with some authors stipulating that they are unacceptable for scientific research on energy intake [55]. Therefore, the energy gap in Fig. 2 should be viewed with caution as this is likely an artefact of participants underreporting energy intake. While a common limitation with food diaries [54], our findings also demonstrate this issue could be exacerbated with increased participant burden (i.e. with a longer measurement period) as participants reported consuming fewer calories from day 1 to day 7 (order effect, *P* = 0.01). It is perhaps advisable at this stage to avoid making inferences about energy balance when equating self-report and objectively measured data. While objective measures of energy intake are beginning to emerge (i.e. digital photography, chewing and swallowing monitors [56, 57]), these are in the early stages of development and future work is required to develop these tools. Considering this, it is important to describe measurement variability when using the next best available method that is commonly used to capture habitual free-living energy intake and is accessible for clinicians.

Researchers might be interested in reliably measuring macronutrient composition as the amount of fat and carbohydrate consumed has previously been associated with body composition outcomes in persons with SCI [53]. In keeping with the able-bodied literature [58, 59], it is clear that a longer period of time is required to estimate dietary macronutrient intake (i.e. 8–24 days) compared to total energy intake (i.e. 4 days). However, it is unclear why our data predicts a greater measurement period to reliably quantify dietary fat intake (24 days) compared to carbohydrate (10 days), protein (8 days) and alcohol (9 days). This might, in part, be related to the fact that we have modelled these data on a 7-day data collection, with a loss of predictive precision beyond 7-days. Previous research has also demonstrated differences between specific macronutrients, with dietary fat requiring longer measurement periods than other macronutrients in Japanese adults [60] and in rural women living in a developing country; fat (188 days), protein and carbohydrate (8–23 days) [61]. Factors intrinsic to persons with SCI, such as physical barriers (e.g. transport to shops and supermarket store shelving), environment (e.g. hospital food), functional challenges (e.g. problems encountered when preparing food) and social factors (comfort food provided by family/friends) [62] could either limit dietary diversity or contribute to a highly variable diet. These factors will ultimately impact appropriate measurement duration. Therefore, if interested in macronutrient composition rather than total energy intake per se*,* researchers should consider using longer measurement periods that include both week and weekend-days, particularly as protein and alcohol intake significantly fluctuate between days (Table 3). Future research should assess the influence of potential intrinsic barriers and motives on dietary behaviour and macronutrient intake in persons with SCI.

Sex and injury level were significant covariates for numerous physical activity outcomes from the linear mixed model analysis. Participants with lower spinal cord injury levels (i.e. greater function) were significantly (*P* < 0.04) more active and less sedentary. There were relatively few females (*n* = 6) in the sample compared to males, possibly reflecting volunteer bias or representing the true incidence of SCI in the wider population (i.e. males are four times more likely to sustain a SCI [63]), meaning any comparisons between sexes are likely underpowered. Future studies should aim to include more female participants, particularly as these results suggest they may be more inactive than their male counterparts. Independently, both energy intake and expenditure are reduced in individuals with SCI compared to the general population [51, 64]. This is an interesting observation considering a recent study has demonstrated low energy flux, not surplus energy, predicts future increases in body fat [65]. Low energy flux seems a plausible hypothesis to explain the heightened rates of obesity in individuals with SCI [2].

A notable limitation of this study is the relatively small sample size, which can be a common occurrence when working with persons with physical disabilities due to challenges with participant identification and recruitment [66]. Besides being one of the larger physical activity datasets in this population, data were also captured using an individually calibrated multi-sensor device that has been validated for use in individuals with SCI [24]. Moreover, this is one of the first papers to explore novel physical activity dimensions (i.e. objectively sedentary behaviour) in persons with SCI, while also providing concurrent estimates of energy intake. As previously recommended [67], we have incorporated multiple statistical tests to fully understand the issues underlying the calculation of an appropriate monitoring timeframe for energy intake and expenditure outcomes. Future research should assess variability over a greater number of days, weeks or even across seasons, although this will likely impact on participant compliance and monitor wear time, which the current results demonstrate have independent effects on reliability.

## Conclusions

This is the first study to demonstrate the variability of energy intake and expenditure components in this population and propose suitable measurement durations required to achieve acceptable reliability in numerous outcome measures. While it is advisable that a 7-day cycle is optimal, capturing all day-to-day variation and weekly behaviours, this is not always possible. Wear time and participant burden played a noticeable role in the quality of energy expenditure and intake data, respectively. Therefore, minimising the number of monitoring days to the reliable lower limit will intuitively have a positive impact on these factors, as well as increasing turnover of expensive monitoring devices in the field. These findings reveal important implications that may inform future study designs and data reduction strategies in this population.

## Abbreviations

CIs: Confidence intervals
CVs: Coefficients of variation
DIT: Dietary induced thermogenesis
ICC: Intra-class correlation
METs: Metabolic equivalents
MVPA: Moderate-to-vigorous physical activity
PAEE: Activity energy expenditure
PAL: Physical activity level
RMR: Resting metabolic rate
SCI: Spinal cord injury
TEE: Total energy expenditure
